# Differences in DNA methylation status explain phenotypic variability in patients with 5p− syndrome

**DOI:** 10.1186/s13104-024-06734-7

**Published:** 2024-04-29

**Authors:** Vanessa Tavares Almeida, Samar N. Chehimi, Gleyson F. S. Carvalho, Yanca Gasparini, Amom M. Nascimento, Lucas L. Vieira, Beatriz M. Wolff, Marília M. Montenegro, Leslie D. Kulikowski

**Affiliations:** Laboratorio de Citogenomica, Departamento de Patologia, Faculdade de Medicina, Universidade de Sao Paulo, PAMB, 2º Floor, Block 12, Room 07, Dr. Eneas de Carvalho Aguiar Avenue, 155, Cerqueira Cesar, Sao Paulo, 05403-000 Brazil

**Keywords:** Cri du chat syndrome, DNA methylation, Rare diseases

## Abstract

Cri Du Chat syndrome, or 5p− syndrome, is characterized by a terminal or interstitial deletion on the short arm of chromosome 5 that causes variable clinical manifestations, including high-pitched cry in newborns, delayed growth, and global development. Different cytogenomic rearrangements, family history, and environmental factors may hinder the genotype–phenotype association. Thus, the phenotypic variability of this syndrome may not be limited only to variations in gene structure, such as deletions and duplications. It is possible that other mechanisms related to the activation or inactivation of promoters and/or exons of actively transcribed genes, such as DNA methylation are involved. Therefore, we studied the genome-wide methylation status profile of peripheral blood samples from fifteen patients with Cri du Chat Syndrome and nine control samples through the array method to look for Differentially Methylated Regions. We found that Differentially Methylated Regions outside the 5p region are mainly associated with regulating gene transcription, splicing, and chromatin remodeling. Most biological pathways are related to transcription, histone and chromatin binding, spliceosome and ribosomal complex, and RNA processing. Our results suggest that changes in the 5p region can cause an imbalance in other chromosomal regions capable of affecting gene modulation and thus explain the phenotypic differences in patients with 5p−.

## Background

Cri Du Chat syndrome (CdCS), also known as 5p− (minus) syndrome, is characterized by partial deletion (terminal and/or interstitial) of the short arm of chromosome 5 [7] and is considered a rare syndrome. The incidence is 1 in 15,000 and 50,000 live births [10].

Patients with this genetic syndrome may present varied clinical characteristics such as high-pitched cry at birth, low weight, microcephaly, ocular hypertelorism, hypotonia, micrognathia, low-set ears, prominent nasal bridge, neurological and behavioral alterations, delay in growth and development [7].

Some regions have already been identified as being related to the SCDC phenotype, for example, region 5p15.2 for intellectual disability and facial dysmorphisms, region 5p15.33 for characteristic crying, and region 5p15.32 for language delay [3, 7]. However, the high clinical variability in these patients poses a considerable challenge to understanding the mechanisms that lead to these differences.

DNA methylation involves adding a methyl group (CH3) to carbon 5 of the pyrimidine ring of the nucleotide cytosine. This mechanism generates changes in chromatin and can occur in several places within a gene. However, methylation is more frequent in the “CpG islands” (cytosine-guanine), a region of the genome located in promoters and/or exons of actively transcribed genes, thus being able to control gene expression.

Given this, the genetic alterations of SCDC may not be limited to changes in gene structure alone. Specific alterations may be related to the activation or inactivation of genes by mechanisms such as DNA methylation.

In this manuscript, we report a deep analysis of DNA methylation in a cohort of fifteen Brazilian Cri du chat patients and nine control samples through the array method to correlate the findings to the very variable phenotypic expressivity, not yet wholly understood for this disease, highlighting the individual phenotype of patients.

## Main text

DNA methylation was measured from blood samples of fifteen Cri du chat patients and matched controls by Illumina EPIC arrays. When comparing the data obtained from the array of the SCDC group with the Control group, considering the criterion *p* values < 0.05, that indicates the quality of data is satisfactory. We got a list of differentially methylated regions (DMRs) that are arranged in order of relevance (most significant for least significant about the difference in methylation).

Based on the analysis of the DMRs between case groups versus control groups, we verified that most probes are located on the 5p chromosome, which was expected due to the 5p− syndrome. We had 986 DMRs returned, but we decided to filter only the first 30 DMRs, due to their relevance to the p value. Among the 30 DMRs, 10 DMRs are located outside the 5p region, as described in Table 1.

**Table 1:** Description of the ten significant DMRs outside the 5p region

Chromosome/region	Gene	Description
8p12	*WRN*	Role in maintaining genome stability and in DNA repair, replication, transcription, and telomere maintenance
	*PURG*	Important in controlling DNA replication and transcription
5q35.3	*MIR4638*	Involved in post-transcriptional regulation of gene expression
6q13	*DPPA5*	Involved in the maintenance of pluripotency of embryonic stem cells
16q22.2	*DHODH*	A mitochondrial protein is located on the outer surface of the inner mitochondrial membrane
2p13.3	*SNRNP27*	It plays important role in pre-messenger RNA splicing, facilitating the recognition and selection of splicing sites
7p15.2	*HOXA3/5/6*	It encodes DNA-binding transcription factors that can regulate gene expression
14q32.33	*MTA1*	Involved in the regulation of transcription due to chromatin remodeling
22q12.3	*TXN2*	Important in regulating mitochondrial membrane potential and protecting against oxidant-induced apoptosis
12q24.31	*BCL7A*	Chromatin Remodeling Complex
19q13.12	*WIZ*	It has DNA-binding transcription factor activity and transcription co-repressor binding activity

We also perform functional analyses of pathway enrichment biological (ontologies) based on the complete list of differentially methylated probes (DMPs) obtained in the comparison between groups, considering the criterion *p* < 0.05. We aimed to understand which pathways were associated with the DMPs returned from the comparison, and we selected the pathways that contained the highest numbers of DMPs. We used information from the Kyoto Encyclopedia of Genes and Genomes (KEGG) database and the Gene Ontology (GO) consortium. Table 2 shows the main biological pathways involved in DMPs.

**Table 2:** Description of the main biological pathways based on DMPs

Biological pathway	Data Bank	N° DMPs	p-value
Spliceosome	KEEGG	59	0.0082
Translation regulatory activity	GO—MF	53	0.0058
Transcription factor binding	GO—MF	147	0.0004
Transcriptional co-repressor and co-regulatory activity	GO—MF	182	0.0028
RNA binding	GO—MF	590	0.0005
Binding of the ribonucleoprotein complex	GO—MF	58	0.0034
Pre-messenger RNA binding	GO—MF	21	0.0007
Nucleic acid binding	GO—MF	1281	0.0011
Histone deacetylase binding	GO—MF	51	0.0019
Chromatin binding	GO—MF	213	0.0001
Spliceosome complex	GO—CC	76	0.0009
Ribonucleoprotein complex	GO—CC	253	0.0013
Protein DNA complex	GO—CC	70	0.0005
Nucleus	GO—CC	2352	0.0006
Nucleoplasm	GO—CC	1432	0.0013
Complex containing nuclear protein	GO—CC	419	0.0011
Nuclear chromosome	GO—CC	84	0.0008
Condensed chromosome	GO—CC	94	0.0004
Chromosome	GO—CC	602	0.0009
Telomere maintenance via telomere trimming	GO—BP	12	0.0003
Ribosomal RNA metabolic process	GO—BP	91	0.0002
RNA splicing	GO—BP	162	0.0001
RNA Processing	GO—BP	179	0.0004
Non-coding RNA processing	GO—BP	140	0.0001
Messenger RNA processing	GO—BP	179	0.0002
Metabolic process of messenger and non-coding RNA	GO—BP	251	0.0001
Chromosomal organization	GO—BP	307	0.0006
Biogenesis of cellular components	GO—BP	1029	0.0005

KEGG and GO (databases). Pathways: MF (Molecular Function), CC (Cellular Component) and BP (Biological Process). Nº DMPs is the amount of DMPs found in that pathway

The DMRs outside the 5p region are primarily associated with regulating gene transcription, splicing, and chromatin remodeling (Table 1). In addition, most biological pathways found are related to transcription, histone and chromatin binding, spliceosome and ribosomal complex, and RNA processing (Table 2). This suggests that the 5p deletion can cause an imbalance in other genomic regions beyond the 5p− chromosome. This methylation difference in 5p− patients versus the control group would be able to modulate the phenotype and, thus, explain the phenotypic differences in patients with this syndrome.

**Fig. 1:**
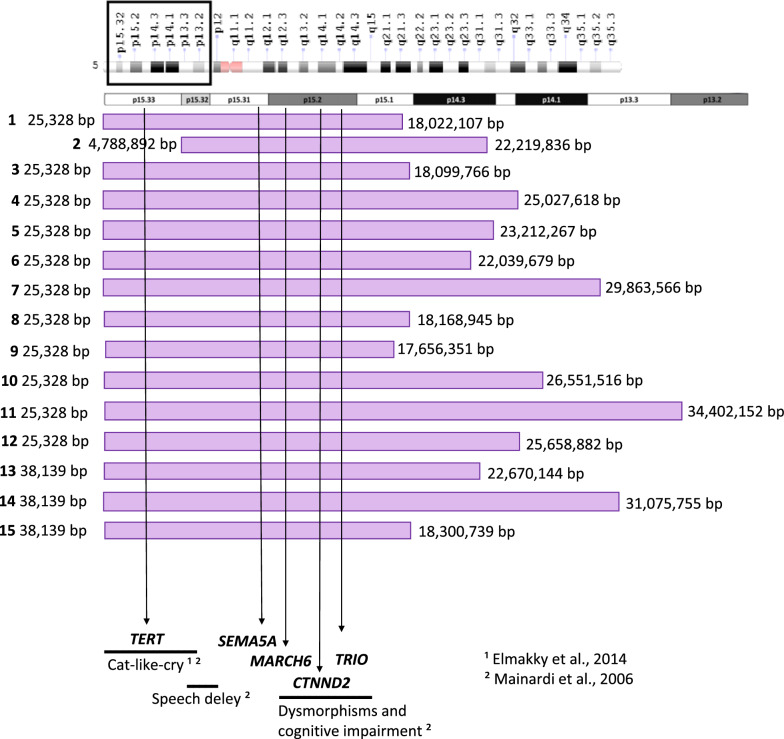
Representation of the extent of the deletion 5p of each patient, indicated by the horizontal lilac bars, and breakpoints in basepairs detected through genomic array. *TERT, SEMA5A, MARCH6, TRIO,* and *CTNND2* are marked with vertical arrows. The arrows have presented the genotype–phenotype relationships from the data of Elmakky and Mainardi. *bp* basepairs, *p* short arm, *q* long arm

**Fig. 2:**
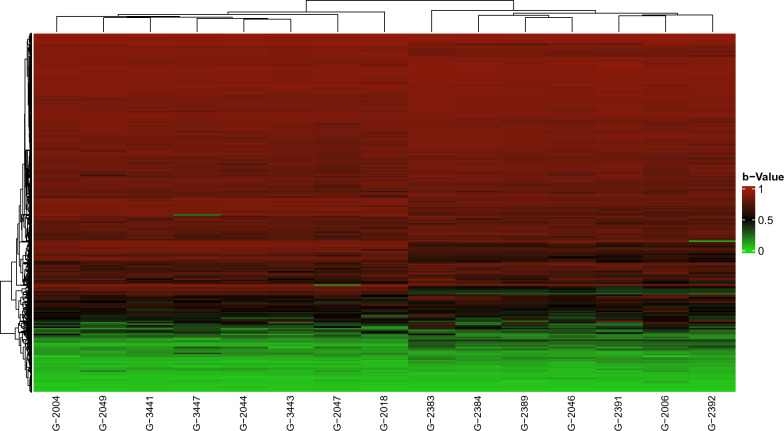
Heatmap of the breakpoint of the 15 patients with CdCS. The probes represented by each line follow the genomic coordinate sequence of the region 5p of each patient. The b-value was used for graphical representation. b-Value: (β); Green color: probes with b-values ≤ 0.3 are representative of hypomethylation. Black color: b-values between 0.31 and 0.7 are representative of hemimethylation; Red color: b-values ≥ 0.71 are representative of hypermethylation. Indicating status without methylation, methylation on only one DNA strand, and complete methylation on both DNA strands. The heatmap groups patients by similarity

We also performed an individual analysis of the breakpoints defined by the genomic array (Illumina 850 K—Fig. 1), with 1 Mb flanking for each patient, returned chromosome 5p DMPs that follow the sequence of the genomic coordinates of the deleted critical region (5p−). In this way, for each patient, it is possible to verify the difference in the methylation status by the heatmap (Fig. 2).

The second figure shows the difference in the methylation status of the remaining allele (copy remainder of the 5p deletion). The design of the EPIC methylation experiment [11] has a probe for the methylated allele and a probe for the unmethylated allele. Thus, when there is only one allele (one copy), this allele is seen as homozygous in the results. Given this, there is significant heterogeneity in the status of methylation within the deleted region, with small segments hypomethylated, followed by others with hemimethylation and others with hypermethylation, as well as in regions with several copies within the normal range (two copies).

**Fig. 3:**
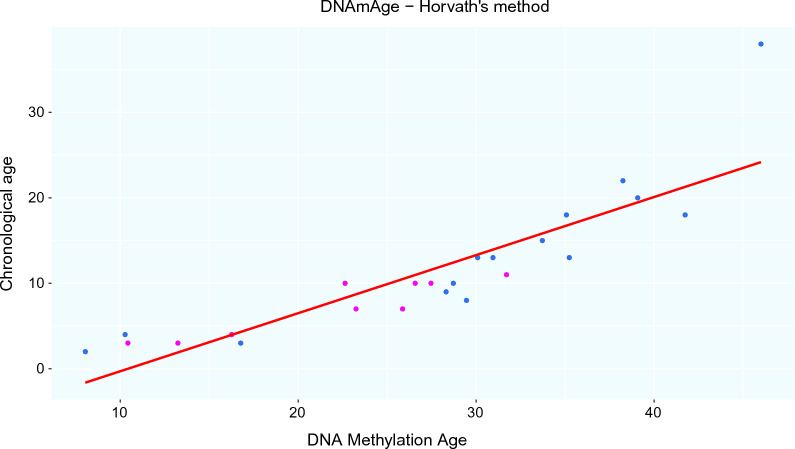
Scatter plot with the distribution of chronological ages (y-axis) and biological age estimation through DNA methylation markers (DNAmAge) (x-axis). Blue color: patients with CdCS. Lilac color: control samples

We analyzed the DNAmAge using Horvath’s method [5] of 5p− patients versus a control group. This method reported a multi-tissue age estimator, referred to as an epigenetic clock, that uses site-specific DNA methylation patterns of 353 CpGs. This methylation assessment is based on previously studied genes linked to senescence. We observed a slightly significant association of acceleration of biological age in patients with Cri du Chat syndrome about controls, as observed in Fig. 3 and Table 3.

**Table 3:** Estimation of DNAmAge based on methylation markers

Patients/controls	Gender	Chronological age	DNAmAge	ageAcc	ageAcc2	ageAcc3
1 (G-2006)	F	22	38.25	16.25	− 0.862	− 2.328
2 (G-2046)	F	13	30.084	17.084	1.112	− 1.675
3 (G-2383)	F	2	8.038	6.038	− 8.538	− 1.701
4 (G-2384)	F	8	29.455	21.455	6.117	4.367
5 (G-2389)	M	18	35.071	17.071	0.465	− 0.316
6 (G-2391)	F	13	35.233	22.233	6.261	6.439
7 (G-2392)	M	38	46.004	8.004	− 11.136	− 6.806
8 (G-2004)	F	4	10.273	6.273	− 8.557	− 0.873
9 (G-2044)	F	9	28.311	19.311	3.846	− 1.026
10 (G-2049)	F	13	30.945	17.945	1.973	0.212
11 (G-2018)	F	15	33.723	18.723	2.497	1.788
12 (G-2047)	F	10	28.719	18.719	3.127	3.611
13 (G-3443)	M	20	39.078	19.078	2.219	1.894
14 (G-3441)	F	3	16.769	13.769	− 0.935	− 2.048
15 (G-3447)	F	18	41.745	23.745	7.139	9.32
ctrl 1 (V-13)	M	10	26.575	16.575	0.983	1.606
ctrl 2 (V-34)	F	3	10.427	7.427	− 7.276	− 3.623
ctrl 3 (V-58)	F	3	13.24	10.24	− 4.464	− 8.357
ctrl 4 (V-65)	F	4	16.261	12.261	− 2.569	0.196
ctrl 5 (V-82B)	F	10	27.461	17.461	1.869	− 0.492
ctrl 6 (V82A)	F	10	22.635	12.635	− 2.956	− 4.397
ctrl 7 (V85B)	M	7	25.867	18.867	3.656	2.001
ctrl 8 (V-85A)	M	7	23.249	16.249	1.038	0.175
ctrl 9 (V-95)	M	11	31.706	20.706	4.987	2.029

*ageAcc* the difference between DNAmAge and chronological age, *ageAcc2* residuals obtained after chronological age regression and DNAmAge, *ageAcc3* residues obtained after chronological age regression and DNAmAge adjusted for cell count blood

## Limitations

We emphasize that the epigenetics of neuronal cells and of any other cell is not the same as the epigenetics of lymphocytes. Our study has limitations and is dedicated to the epigenetic study of peripheral blood lymphocytes. Studies with neuronal cells are still scarce in the literature. We relate the mapped regions with the clinical phenotype, where the findings provide perspectives for future studies, which is a model of epigenetic approach for the study of differentially methylated genes.

### Patient recruitment and illumina EPIC arrays

We analyzed fifteen peripheral blood DNA samples from patients with Cri du Chat Syndrome that geneticists followed at the Unit of Clinical Genetics, Instituto da Criança, Hospital das Clinicas, Universidade de Sao Paulo (ICr-HCFMUSP), Brazil, and nine control samples were previously genotyped and classified as normal according to their genomic profile. DNA methylation was measured on Illumina EPIC arrays. This platform was chosen since it interrogates around 853,307 methylation sites per sample, distributed in enhancer, promoter, and intergenic regions.

### Data analysis

The analysis was performed using the R programming language (Rstudio v4.0.2) following the pipeline proposed by Maksimovic [8] with modifications. This article provides examples of the steps involved in analyzing methylation array data using packages from the Bioconductor repository [6].

The sequence of steps analyzing the array data from methylation using the R programming language was sample preparation, data quality control, normalization, filtering, differential methylation analysis, Functional analysis, mapping of methylation at breakpoints of patients with 5p−, and determination of DNAmAge.

For the preparation of the samples, we performed the internalization of the raw data.idats, the identification of these data, and separation by case/control groups based on a previously prepared sample sheet. For data quality control, we evaluated the average detection of *p* values, representative of the fluorescent signal intensity of each probe. Thus, samples with an average greater than 0.05 are then excluded from the analysis. The normalization step aims to minimize intra- and inter-sample variation using the preprocessQuantile method (suitable for single-tissue datasets). After normalization, information on the intensity of methylated and unmethylated probes was converted into M and β (beta) values, along with the associated genomic coordinates.

Some filtering was done to remove probes from below performance (based on *p* value); X and Y chromosome probes; probes of SNPs at the same CpG site and cross-reacting probes that are regrettable to map to multiple sites in the genome.

After these steps, the methylation analysis was carried out differential between patients with SCDC based on phenotypic characteristics and comparing case/control group. We used M values for statistical calculations, with Student’s t test, for example, and subsequent measurement of methylation status and identification of potential probes and regions differentially methylated (DMRs and DMPs). The β value was used for the final graphical representation of the data obtained.

For the functional analysis of genetic ontologies (enrichment of biological pathways), we used the database with genomic and functional information: Kyoto Encyclopedia of Genes and Genomes—KEGG (https://www.genome.jp/kegg/), and the consortium: Gene Ontology—GO (http://www.geneontology.org/), which provides information to describe biological processes (BP), molecular functions (MF) and cellular components (CC). These functional analyses were based on the complete lists of DMPs obtained in the differential methylation analyses, to understand the biological processes in which these DMPs may be involved. All functional pathways with an adjusted *p* value of less than 0.05 were defined as enriched.

Finally, the determination of biological age through DNAmAge was performed using the method of Horvath [5], who reported a multi-tissue age estimator, referred to as an epigenetic clock, which uses patterns of DNA methylation of specific sites of 353 CpGs. This methylation assessment is based on previously studied genes linked to senescence.

Thus, Excel files containing information on DMRs, DMPs, and ontologies based on differential and functional methylation analysis were generated. Files were generated for graphical visualization of the steps performed, such as a Heatmap for mapping the methylation status, and a Scatter plot for the distribution of chronological/biological ages.

## Discussion

The gene expression is controlled by regulatory elements that may be distant and distributed along the chromosome or, in some cases, on other chromosomes [9, 12]. In this way, we chose not to limit to investigating only the 5p− breakpoint, but, in general, covering all meaningful information regardless of your location.

**Fig. 4:**
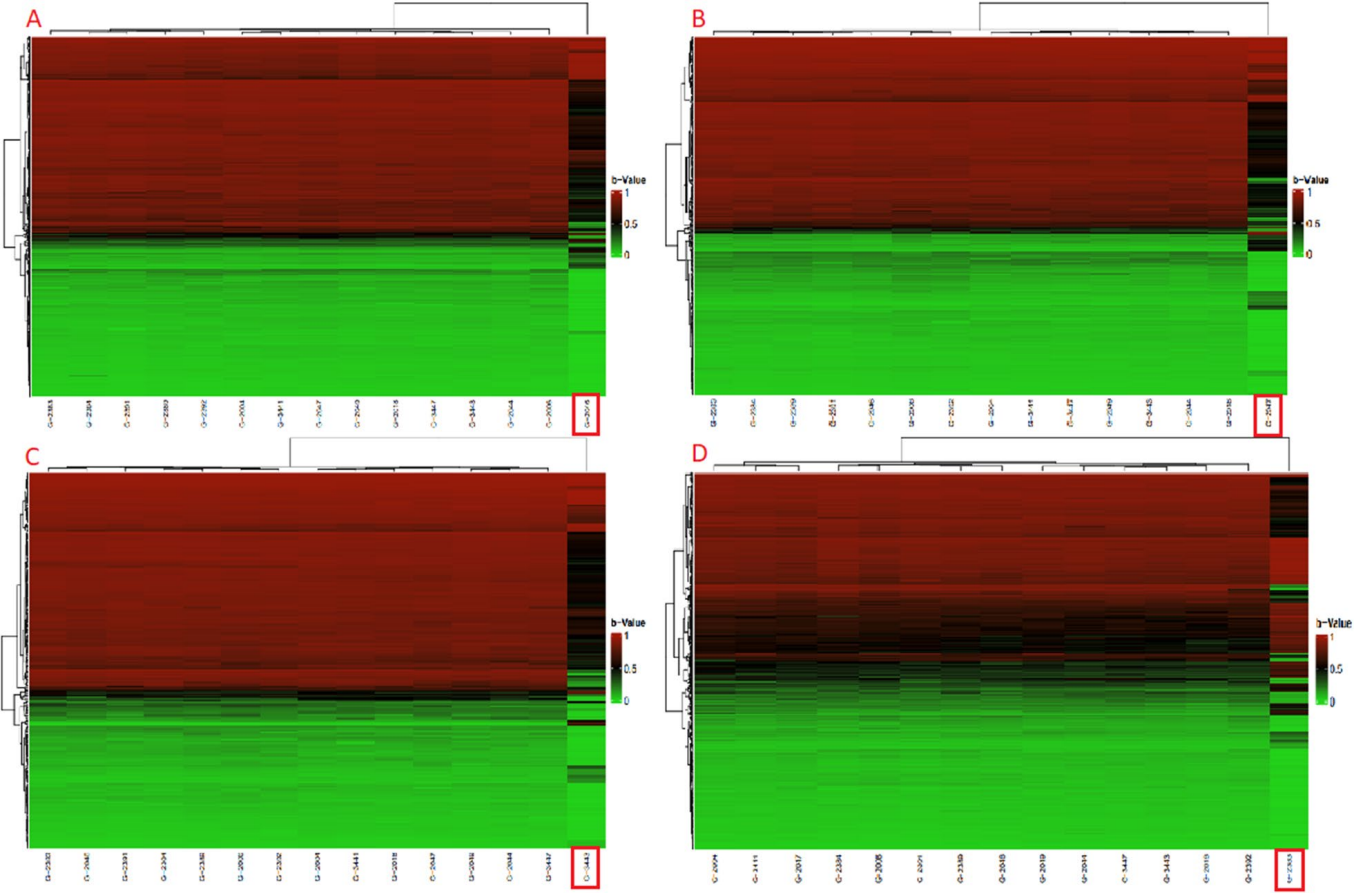
Comparison of the methylation profile between patients with a specific phenotype versus patients without the phenotype. Highlighting in red color indicates the patients discussed in this study. Patient 2 (**A**); 12 (**B**); 13 (**C**) and 3 (**D**)

We identified that some patients have unique characteristics compared to others (Table 4). For the phenotypic differences, we analyzed the DMPs from the comparison between patients with a phenotype versus patients without the phenotype, to identify an epigenotype-phenotype relationship (Fig. 4).

**Table 4:** Phenotypes of patients with 5p−

Phenotypes	Patients														
	1	2	3	4	5	6	7	8	9	10	11	12	13	14	15
high-pitched cry weak/strong	+	++	+	+	+	+	+	+	+	+	+	+	+	+	+
NPMD	+	+	+	+	+	+	+	+	+	+	+		+	+	+
Ocular hypertelorism	+	+	+	+	+	+		+	+	+	+	+		+	+
Microcephaly			+	+	+	+	+	+	+	+	+	+	+	+	+
Short Filter	+	+	+		+	+	+		+	+	+	+	+	+	+
Irritability		+		+		+		+	+	+		+	+	+	+
Self aggression	+	+		+	+	+	+	+	+		+	+			
Sharp face	+						+	+	+	+		+	+	+	+
Epicanthal folds	+	+	+	+				+	+	+			+		+
Obsessive–compulsive disorder	+			+		+	+		+	+	+	+		+	
Prominent nasal bridge	+					+	+		+	+	+	+	+		
High palate	+	+		+		+	+	+	+				+		
Microretrognathia	+		+		+	+	+		+	+	+				
Autistic traits	+	+			+	+	+			+	+		+		
Clinodactyly			+	+	+	+	+		+				+		+
Hyperactivity	+	+		+	+			+		+	+				+
Cleft lip	+	+	+	+	+	+							+		
Strabismus	+			+		+	+	+			+		+		
Heteroaggression	+	+		+		+		+	+	+					
Gastroesophageal reflux	+	+						+	+			+	+		
Palpebral fissures	+	+				+			+			+			
Facial asymmetry	+			+			+			+			+		
Anteverted ears			+				+						+	+	+
Scoliosis		+				+				+	+		+		
Dysplastic ears	+									+	+		+		
Brain malformation			+		+				+		+				
Bitemporal narrowing	+			+							+	+			
Hypotonia				+				+				+	+		
Sandal gap							+	+				+			
Down curved mouth									+	+	+				
Rounded face			+								+		+		
Hyperpigmentation		+						+			+				
Crooked foot		+									+				
Short neck			+								+				
Nasal voice	+											+			
Myopia							+	+							
Prominent columella										+	+				
Hypertrichosis			+												
Seizures													+		
brachydactyly										+					
Syndactyly							+								
Polydactyly			+												
Craniostenosis		+													

+ Present of the phenotype

Patient 2 (A) does not have a high-pitched cry at birth. According to Mainardi and Elmakky [3, 7], the 5p15.33 region is related to high-pitched crying, a region the patient has not deleted. The absence of this feature may be due to the patient having 2 copies of this region. In Fig. 4A it is possible to notice a significant difference in regions with hemimethylation (methylation in only one allele, black color), which are in several regions of the genome. For this comparison, we found a differentially methylated probe in the typical high-pitched crying region (5p15.33), cg23989709 probe, and *SLC12A7* gene, located in the shore region (1 to 2,000 base pairs away from the CpG island).

Patient 3 (D) is the only one with hypertrichosis located on the back and arm. For the DMRs and DMPs analyzed, it was impossible to directly correlate with the genes related to hypertrichosis available in the literature. Even not finding a specific gene related to the hypertrichosis phenotype, other genes not yet described in the literature may be involved. Furthermore, looking at the genome in general (Fig. 4), and not just focusing on a specific gene or region for a phenotype, we see a significant difference that may explain some phenotypes that are not limited to a gene or region.

Patient 12 (B) is the only one without neuropsychomotor developmental delay (NPMD), in addition, she is one of the only patients who understands commands, forms simple sentences, knows colors, letters, and numbers, and eats and cleans by herself. The other patients have more difficulties in these activities. CdCS studies highlight that NPMD is a common feature of this syndrome. Furthermore, we emphasize that all patients have intellectual disability (ID), even if in some cases it is mild to moderate and in other cases more severe.

The cg15895391 probe, which is hypomethylated in patient 12 (B) and hypermethylated in the other 5p− patients, is located at 5p15.2, has the TRIO gene that has been linked in the literature with intellectual disability and neurobehavioral problems, including autistic traits and attention deficit hyperactivity disorder (ADHD) [13]. The patient does not have autistic traits or hyperactivity. All patients lost a copy of this region, therefore, the information on methylation status refers to the remaining allele. As this gene is dose-sensitive, its transcription may be different in patients based on the copy of the remaining allele. The cg14507238 probe, which is also hypomethylated in the patient and hypermethylated in the other 5p− patients, is located at 12p13.31, has the *ANO2* gene, which is a calcium-activated chloride channel, and has been described in several brain regions with evidence of a role of chloride-dependent modulation in the olivo-cerebellar system that may be important for cerebellar-dependent motor coordination and proper learning [1].

Patient 13 (C) suspected Prader–Willi syndrome (PWS) due to the patient having binge eating, lack of satiety, and hypotonia. The patient does not have obesity or hypogonadism. In the genomic array, we found no CNV on chromosome 15, and no region of homozygosity (ROH) in the 15q11 region, a critical region for PWS. However, we found a differentially methylated region in this patient compared to other patients 5p− (chr15:25334879–25334988), 15q11.2 region, *SNHG14* gene. And a differentially methylated probe (cg18909847), 15q11 region, *SNORD116* gene, is in OpenSea (4000 base pairs away from the island).

Although PWS is considered a contiguous gene syndrome, based on deletions and uniparental disomy, the lack of expression of only one non-coding RNA transcript of the *SNURF-SNRPN/SNHG14* gene may be one of the causes of PWS. Furthermore, small atypical deletions in the paternal *SNORD116* gene are related to most clinical phenotypes of PWS [2]. Given this, it is important to emphasize that, although there is information about the regulation and expression of certain genes and transcripts derived from the 15q11-q13 locus, there is still much to be understood about their true contribution at the molecular level to clinical features of PWS. We believe that this difference in methylation may clarify some phenotypic characteristics, however, further studies using other omics technologies (transcriptomics and proteomics) should be carried out.

In a recent study by Holland [4], there is a hypothesis related to the loss of one or several dose-sensitive genes on chromosome 5 that may cause changes in developmental programs during embryo development, possibly contributing to the development of phenotypes in the patient, that is, whether a dose-sensitive gene has differences in methylation related to epigenetic functions in general, patients can develop a certain phenotype. In our study, mainly comparing cases versus controls, we identified DMRs and biological pathways related to epigenetic functions, which helps us answer this hypothesis.

## Conclusions

It was possible to identify that the methylation profiles of patients with 5p− are different in between, even though some patients have practically the same deletion size. We found that Differentially Methylated Regions outside the 5p region are mainly associated with regulating gene transcription, splicing, and chromatin remodeling. Most biological pathways are related to transcription, histone and chromatin binding, spliceosome and ribosomal complex, and RNA processing, suggesting that changes in 5p− can cause an imbalance in other chromosomal regions capable of affecting gene modulation and thus explain the phenotypic differences in patients with 5p−. The findings of epigenetic modifications improve understanding of the human genome and clarify some epigenetic mechanisms in Cri du Chat syndrome, bringing knowledge about the genetic content of specific regions.

## Data Availability

The datasets used and/or analysed during the current study are available from the corresponding author on reasonable request.

## Abbreviations

DMR: Differentially methylated regions
DMP: Differentially methylated probes
CpG: CG-dinucleotide where the C is commonly methylated
CdCS: Cri du chat syndrome
MF: Molecular function
CC: Cellular component
BP: Biological process
NPMD: Neuropsychomotor developmental delay
ID: Intellectual disability
ADHD: Attention deficit hyperactivity disorder
PWS: Prader–Willi syndrome
ROH: Region of homozygosity
DNAmAge: DNA methylation markers
